# Kinetic ways of tailoring phases in high entropy alloys

**DOI:** 10.1038/srep34628

**Published:** 2016-09-30

**Authors:** Feng He, Zhijun Wang, Yiyan Li, Qingfeng Wu, Junjie Li, Jincheng Wang, C. T. Liu

**Affiliations:** 1 State Key Laboratory of Solidification Processing, Northwestern Polytechnical University, Xi’an 710072, China; 2 Center of Advanced Structural Materials, Department of Mechanical and Biomedical Engineering, College of Science and Engineering, City University of Hong Kong, Kowloon, Hong Kong, China

## Abstract

The comprehensive performance of high entropy alloys (HEAs) depends on the phase selection significantly. However, up to now, investigations of the phase selection in HEAs mainly focused on the thermodynamic equilibrium phase, while kinetic ways of tailoring the phases in HEAs are seldom considered. In HEAs, the kinetics of sluggish diffusion and the numerous possible phases make the kinetics of phase transformation more complex and intriguing. Here, the kinetic effect in CoCrFeNiTi_0.4_ HEAs was investigated to reveal the possibility of controlling phase selection via kinetic ways for HEAs. The σ, γ′ and R phases in the CoCrFeNiTi_0.4_ HEA can be controlled under different cooling rate both in solidification and solid transformation. The theoretical analyses revealed the kinetic effect on phase selection. The method proposed here, tailoring the phases with different kinetic ways, could be used to prepare promising HEAs with very rich composition design.

Conventional alloys always consist of one principal component with several minor elements added to tailor the properties. For a long time, the development of alloys was restricted to this manner. The minor elements are with strict restriction on the concentration to prevent the formation of complex microstructures, especially the intermetallic compounds leading to a fragile mechanical behavior. Different from the traditional alloys, a new class of alloys was proposed by Cantor and Yeh *et al.* in 2004: high entropy alloys (HEAs)^1^,^2^,^3^,^4^,^5^,^6^,^7^. HEAs differentiate from conventional alloys by equal or near-equimolar chemical compositions and attracted a lot of attentions due to their potential applications as well as the extended opportunity of alloy design^8^,^9^,^10^,^11^,^12^,^13^,^14^,^15^,^16^.

Phase selection is a crucial issue in HEAs. In the past decade, great efforts have been made to explore HEAs with thermally stable single solid solution phase. Some of the existing work focused on the extension of Hume-Rothery rules, such as atomic size (δ, γ), electronegativities (∆χ), and valence electron concentration (VEC)^9^,^10^,^13^,^17^,^18^,^19^. Some researchers concentrated on the competition of mixing enthalpy (∆H) and ideal mixing entropy (∆S) to develop criteria of phase stability^3^,^20^,^21^. Moreover, researchers also tried to use CALPHAD method and ab initial calculations to predict phases of HEAs^22^,^23^. These previous efforts are helpful to search for single phase HEAs, however, it was found that only several alloys consist of single solid solution while most HEAs are with multi-phases. For example, HEAs like CoCrFeNiTi_x_^24^,^25^, CoCrFeNiMo_x_^26^ and CoCrFeNiNb_x_^27^,^28^,^29^ consisting of complex microstructures and phases. Intermetallic compounds, like σ, μ, Laves and R phases occurred in these HEAs. Thus methods to control the phases of HEAs are needed for a better performance. The very recent publications^30^,^31^ showed that multi-phase HEAs have better mechanical properties than HEAs with single solid solution phase, but many multi-phase HEAs consist of too many phases and this is bad for mechanical properties. Tailoring the phases in HEAs will bring more metallic materials with high performance.

Thermodynamic ways of annealing are the common way to alter the phases in alloys. Besides the thermodynamic method, kinetics of phase transformation also greatly affects the phase selection, usually by two stages: solidification and solid-state phase transformation. The phase formation in alloys can be well controlled through the phase transformation kinetics^32^,^33^. For example, rapid solidification has been widely used to obtain metallic glass and solid-state phase transformation via thermal treatment has also been often employed to introduce strengthening phases in commercial alloys^34^,^35^,^36^,^37^. In spite of that the kinetics of phase transformation is well known, in HEAs, it deserves more attentions.

Firstly, the phases in HEAs could be very complex because of their concentrated multi-components while phase compositions in traditional alloys are much simpler. There are two solid phases in binary alloys while five different phases at equilibrium state in the CoCrFeNiTi_x_ HEAs^25^. It is complicated but interesting and useful to tailor the phase compositions and their volume fraction in HEAs. Secondly, it is reported that there is sluggish diffusion effect in HEAs^38^. It is intriguing whether this unique phenomenon would affect the kinetic way of phase selection in HEAs.

Thus, as a new class of alloys, the kinetic effect in the phase selection of HEAs is of critical importance in both scientific studies and practical applications. Some reported works also showed that the processing routes have great influence on the phase compositions of HEAs, which actually reflected that the kinetics acted on the phase selection of HEAs^39^,^40^,^41^,^42^. Inspired by kinetics, tailoring phases in HEAs is extremely attractive and promising. Here, by taking the CoCrFeNiTi_0.4_ HEA as example, the methods of rapid solidification after laser remelting and rapid quenching were used to show the kinetic effect of phase transformation.

## Experimental

The CoCrFeNiTi_0.4_ HEA was prepared by arc melting and then cooled in the arc furnace and alloys in this state were noted as as-cast (AC) HEA. Elements of Co, Cr, Fe, Ni and Nb with purity of 99.95% were used as raw materials. Rapid solidification was realized by laser remelting with a laser intensity of 2 KW and a laser spot size of 2 mm. The laser was held on the as-cast CoCrFeNiTi_0.4_ base for 0.3 s forming a laser remelted zone (noted as LR zone) where rapid solidification occurred after removing the laser spot. Some of the as-cast samples were annealed separately at temperatures of 900 °C and 1080 °C for 24 hrs. Two different cooling ways, rapid quenching in water and furnace cooling, were used. The rapidly quenched samples are noted as Q1 (annealed at 1080 °C) and Q2 (annealed at 900 °C), and the samples that were cooled in furnace are noted as FC1 (annealed at 1080 °C) and FC2 (annealed at 900 °C). The CALPHAD method (JMatPro software) was used to calculate the phase diagram, TTT and CCT curves of the CoCrFeNiTi_0.4_ HEA with the TTNi8 data base. Sample phases were identified by an X-ray diffractometer (XRD, Bruker D8 discover), using Co Kα radiation scanning from 25° to 125° with a step of 25° and an exposure of 30 s. The microstructures were characterized by scanning electron microscope (TESCAN VEGA 3) and the chemical compositions were analyzed by SEM energy dispersive spectrometry (EDS). The laser scanning confocal microscope (LEXT-4000) was used to get the macroscopic view of the samples. The hardness measurement was carried out with a Vickers hardness tester (HMV-T2) under a load of 1000 g for 15 s, and an average of at least five different points on each sample was presented.

## Phase selection of CoCrFeNiTi_0.4_ HEA

### Phase selection in solidification process

**Figure 1:**
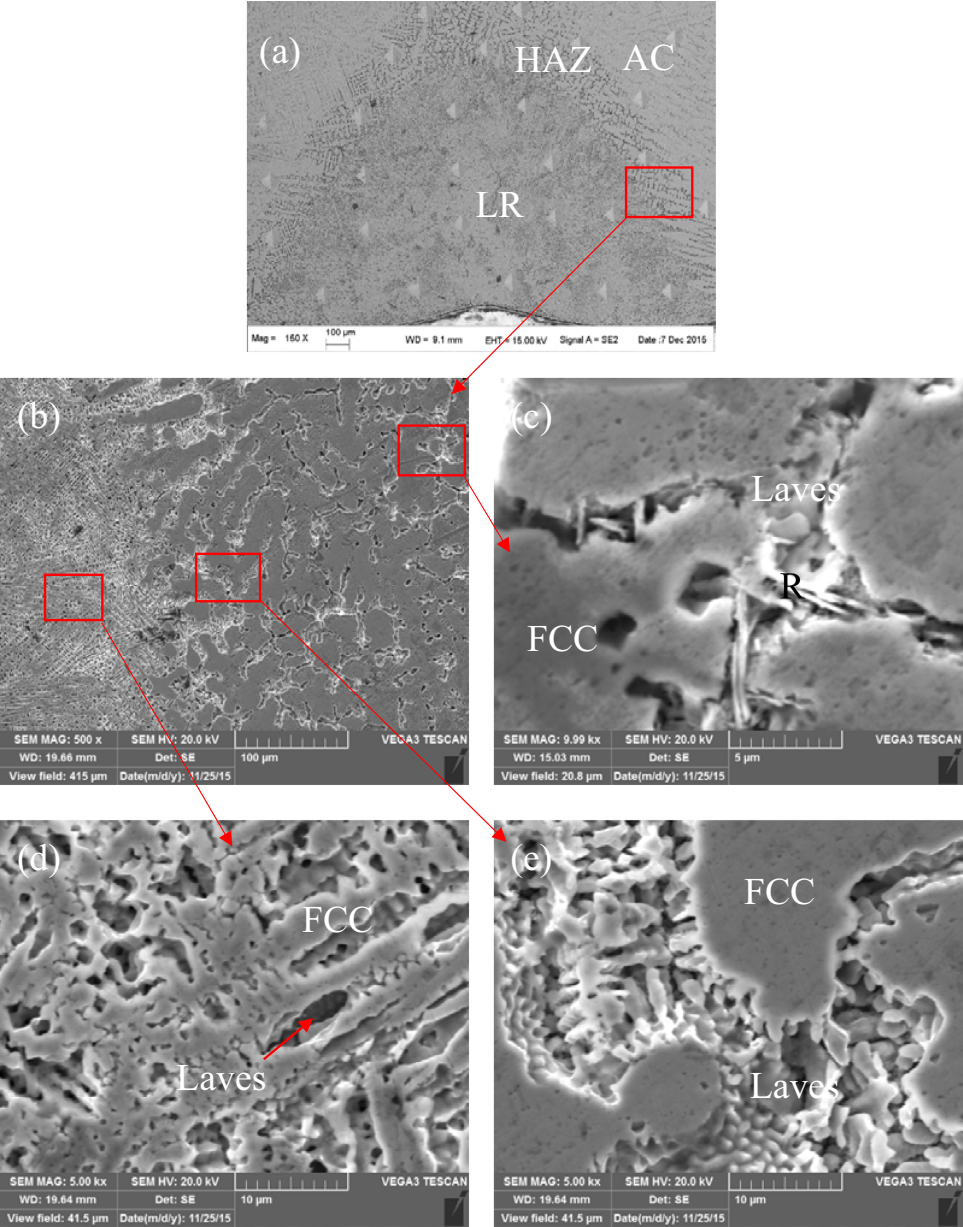
SEM images of the CoCrFeNiTi0.4 HEA; (**a**) low magnification of laser remelted zone and as-cast zone, (**b**) amplification of red rectangle zone in (**a**), (**c**) high magnification of as-cast zone (AC), (**d**) microstructures of laser remelted zone (LR), (**e**) morphology of heat affected zone (HAZ).

**Figure 2:**
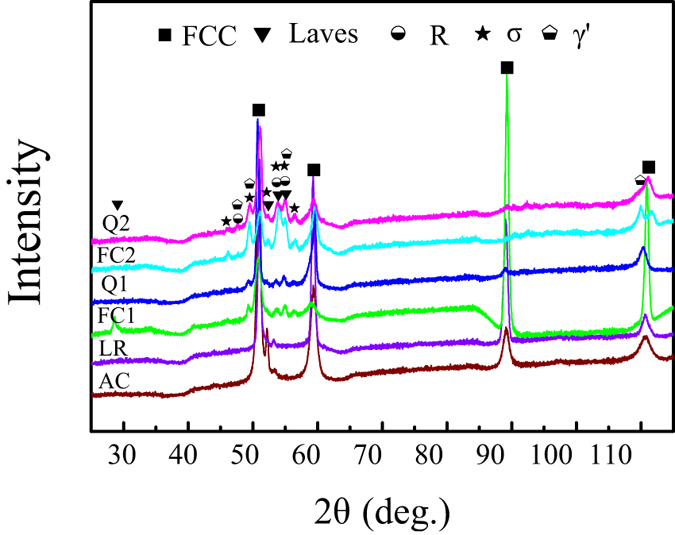
XRD patterns of CoCrFeNiTi_0.4_ HEAs under different conditions, as-cast (AC), heat affected zone (HAZ), laser remelted zone (LR), rapid quenching in water after annealed at 1080 °C (Q1) and 900 °C (Q2), furnace cooling after annealed at 1080 °C (FC1) and 900 °C (FC2).

**Figure 3:**
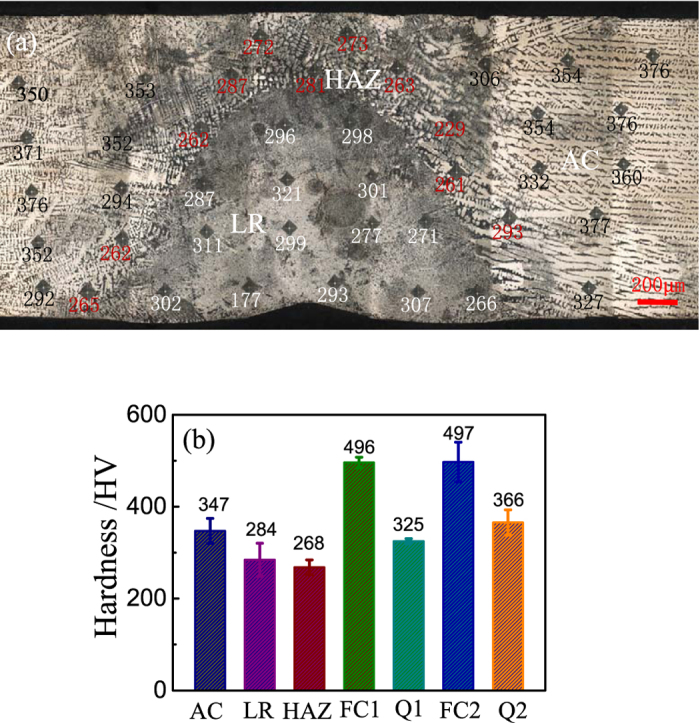
(**a**) Macro morphology of the sample and the corresponding hardness distribution, (**b**) Hardness of the CoCrFeNiTi_0.4_ in different conditions: as-cast (AC), heat affected zone (HAZ), laser remelted zone (LR), rapid quenching in water after annealeda at 1080 °C (Q1) and 900 °C (Q2), furnace cooling after annealed at 1080 °C (FC1) and 900 °C (FC2).

The cooling rate of solidification can heavily affect the phases and their volume fractions of alloys. Figure 1(a) shows the macroscopic view of the laser remelted zone together with the as-cast CoCrFeNiTi_0.4_ HEA. Figure 1(b) presents the microstructures of the AC zone, LR zone and heat affected zone (HAZ) of the CoCrFeNiTi_0.4_ HEA and Fig. 1(c–e) are magnifications of the three zones, respectively. Figure 2 shows the corresponding XRD patterns in different regions or samples. Figure 3 shows (a) the hardness distribution and (b) the statistic results of different zones.

The AC zone shows grained dendrite and platelet-like and white phases in the interdendritic regions. The XRD patterns in Fig. 2 indicated that three different phases can be detected and identified as (Fe, Ni)-type FCC, (Fe, Ti)-type Laves and (Ni, Ti)-type R phases. The EDS results in Table 1 indicated that, the dendrite (primary phase) was composed of almost equimolar Co, Cr, Fe and Ni elements with small amount of Ti, while the platelet-like phase was rich in Ni and Ti elements. Combination of the XRD and EDS results identifies that the primary phase is FCC matrix and the platelet-like R phase and Laves phase coexist in interdendritic regions, agreeing well with reported work^24^,^43^,^44^.

Differently, the LR zone (Fig. 1(c)) shows much finer dendritic microstructures compared with the AC zone. The most important point is that the platelet-like R phase disappeared in the LR zone and the XRD analysis in Fig. 2 also demonstrates that there are only FCC and Laves phases. Therefore, the R phase in the CoCrFeNiTi_0.4_ alloy is inhibited under extremely rapid solidification condition. The variation of the phases is also revealed from the hardness test. Although the dendrite size in LR zone is much smaller than that of the AC zone, the hardness of the LR zone is about 100 HV lower than that of the AC zone, which can only be due to the disappearance of R intermetallic phase.

It is worth noting that a HAZ between AC zone and LR zone occurred and the HAZ is even softer than the LR zone. It is due to that the grain size of HAZ is much larger than that in the LR zone while there is also no R phase in the HAZ. The disappearance of R phase in HAZ should be due to the effect of solid transformation kinetics.

### Phase selection in solid-state phase transformation process

**Figure 4:**
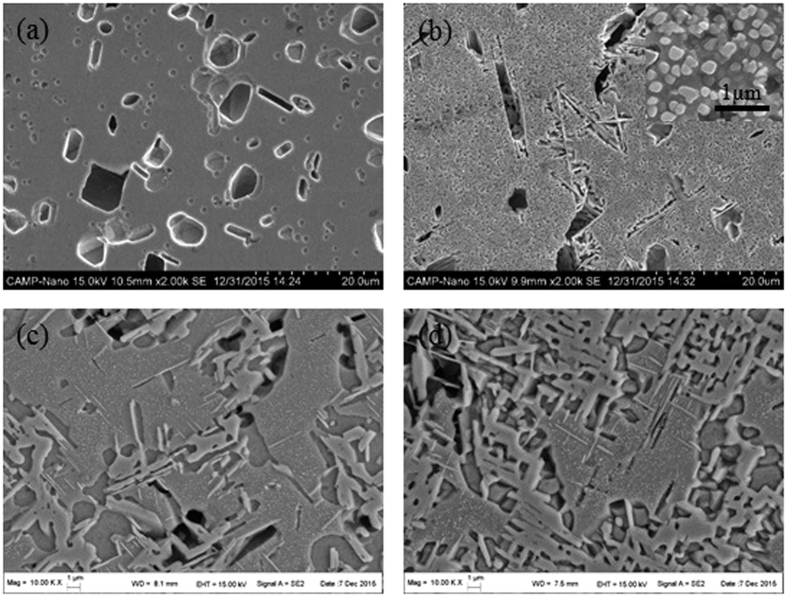
Microstructures of annealed CoCrFeNiTi0.4 HEA; (**a**) 1080oC and quenching, (**b**) 1080oC and furnace cooling, (**c**) 900oC and quenching, (**d**) 900oC and furnace cooling.

Besides the rapid solidification, the effects of solid-state transformation kinetics on the phase selection were investigated via different cooling rate after annealing the CoCrFeNiTi_0.4_ HEA at 900 °C and 1080 °C. The corresponding SEM images are presented in Fig. 4. It shows that, when the CoCrFeNiTi_0.4_ HEA was annealed at 1080 °C and quenched in water (Q1, Fig. 4(a)), only a small amount of platelet-like phase can be found. However, plenty of nano-particles and platelet-like phases showed up when the sample was cooled in the heat treatment furnace (FC1, Fig. 4(b)). The XRD results, as shown in Fig. 2, indicated that Q1 HEA had mainly FCC phase and minor Laves and R phases while the FC1 HEA was composed of γ′, R, σ, Laves and FCC phases. These results illustrated that a high cooling rate during the solid-state phase transformation can prevent the precipitation of γ′ and σ phases.

Besides the cooling rate, the annealing temperature is another key factor that determines the phase compositions. Hence, the similar experiments but with a different annealing temperature was conducted to supplement the above study. Figure 4(c,d) show the microstructures of the CoCrFeNiTi_0.4_ HEA annealed at 900 °C with quenching (Q2) and furnace cooling (FC2), respectively. It is clear that both the alloys have very complex phase compositions. From the SEM images, there are five different phases: dendrite phase, nano-particles precipitated from the dendrite matrix, platelet-like phase in the dendrite matrix, interdendritic phase, and platelet-like phase in the interdendritic zone. The volume fraction of platelet-like phase in the FC2 HEA is larger than that of the Q2 HEA. The XRD and EDS analyses showed that these phases are FCC, γ′, σ, Laves and R phases, respectively. The corresponding hardness tests showed that the HAZ has the lowest hardness and the FC2 HEA has the highest hardness, which is consistent with the phase compositions. These results also demonstrated that the cooling rate remarkably affects the volume fraction of the phases when those phases occurred during annealing process.

### Kinetic effect and tailoring phases in CoCrFeNiTi_0.4_ HEA

Based on the preceding results, the kinetic effect in CoCrFeNiTi_0.4_ HEA can be concluded. The cooling rate of LR HEA is the highest, thus R, σ and γ′ phases are all inhibited in this condition; with the decrease of cooling rate, the R phase shows up in an AC sample. As for the solid-state transformation process, the cooling rate of the Q1 HEA is quite high, and σ phase was restrained under the rapid quenching condition. And actually, the γ′ phase is also inhibited when quenched in water. All the phases can be formed when the cooling rate is sufficiently low, which is similar to the equilibrium condition. The FC1 sample in this study proved this conclusion. If the phases occurred during annealing at the corresponding temperature, as the Q2 and FC2 shown, the cooling rate would not affect the phase selection.

**Figure 5:**
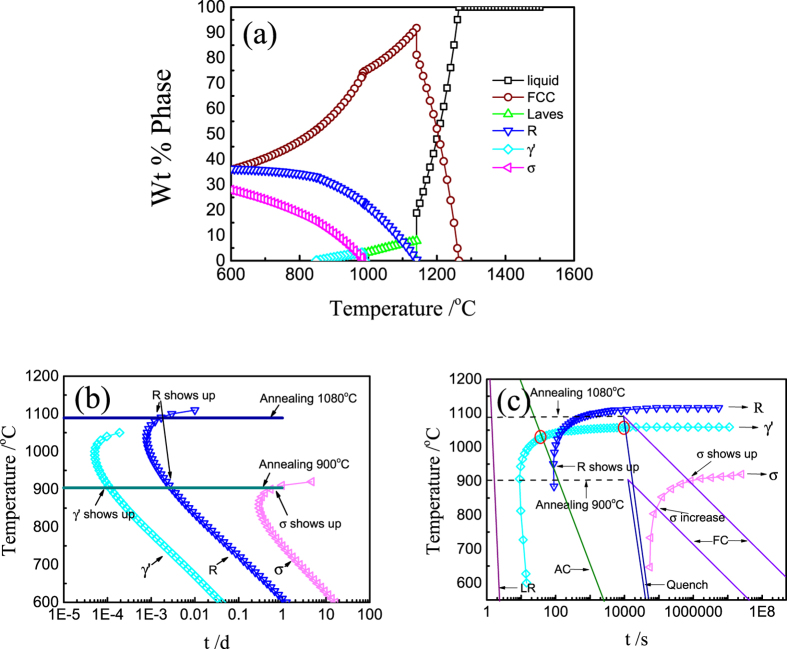
(**a**) Calculated phase diagram of CoCrFeNiTi_0.4_ HEA; (**b**) TTT curve of CoCrFeNiTi_0.4_ HEA from; (**c**) CCT curve of CoCrFeNiTi_0.4_ HEA. All the three figures are obtained from JMatPro software with TTNi8 data base and the eta phase was adjusted to R phase according to the experiment.

Utilizing this kinetic effect, tailoring phases in HEAs can be realized. Figure 5(a) presented the equilibrium phase diagram of the CoCrFeNiTi_0.4_ HEA obtained by thermodynamic calculations. Under equilibrium conditions, the FCC solid phase occurred and then the rest liquid transformed into FCC and Laves phases simultaneously. As the temperature goes down, the FCC and Laves phases will transform into R phase. When the temperature is lowed to 1000 °C, the σ and γ′ phases occur, and the equilibrium phase compositions at room temperatures are FCC, R and σ phases. However, the phase compositions can be controlled using kinetic ways.

The cooling rate of extremely rapid solidification induced by laser remelting is around 1×10^6^ K/s and thus there is no time for the liquid to change its compositions, resulting in the formation of the supersaturated FCC matrix and a small amount of the Laves phase. Table 1 shows that the Ti content of the dendrite in the LR zone is almost equal to the normal compositions, identifying that the extremely rapid solidification process prevented the change of chemical compositions. Due to the abrupt decrease of temperature and the low diffusion coefficient at low temperatures, the supersaturated FCC and Laves phases cannot be transformed into other equilibrium phases and were presented as the metastable phases at room temperatures. In the Continuous Cooling Transformation curve (CCT), as shown in Fig. 5(c), the cooling rate curve did not intersect with any of the phase curves, which also supported the experimental results.

Different from the laser remelting induced rapid solidification, the AC sample was cooled in the electric-arc furnace, and its cooling rate is much lower than that of the LR zone. During the growth of the FCC matrix, the redistribution of the chemical composition occurred and the Ti element was enriched in the residual liquid phase. When the Ti concentration reaches up to a critical level, the Laves phase begins to appear in the liquid. Because of the relatively slow cooling rate and the high diffusion coefficient at temperatures above 1000 °C, there is still time for some amount of R phase to precipitate out. Hence, the AC HEA consisted of FCC, Laves and R phases. The phase selection can also be predicted by CCT (Fig. 5(c)) curve. The cooling rate did not intersect with σ and γ′ curves, thus these two phases were inhibited.

Heat treatment is widely used in commercial alloys and the corresponding theoretical guidelines are presented as Temperature Time Transformation (TTT) curve and Continuous Cooling Transformation CCT curve. Figure 5(b,c) are the TTT and CCT curves of the CoCrFeNiTi_0.4_ HEA, respectively. According to the TTT curve, the alloy is composed of R, FCC and Laves phases when the annealing temperature is higher than 1050 °C and holds at the temperature for a sufficient time. When it is annealed between 920 °C and 1050 °C, the alloy consists of R, γ′, FCC and Laves phases. R, γ′, σ, FCC and Laves phases coexist when the annealing temperature stays between 600 °C and 920 °C. Thus, as shown in Fig. 5(b), the phase compositions in this study should be predicted as FCC, Laves and R at 1080 °C and FCC, Laves, R, σ and γ′ phases at 900 °C, which is consistent with the microstructures and XRD analysis.

Besides the annealing temperature, the cooling rate also plays a crucial role in phase selection. In the CCT curve, the phase precipitates from its matrix when the cooling rate curve intersects with the corresponding phase transformation curve. If the cooling rate is slow enough, all phases can be formed, but an increase of the cooling rate will inhibit the precipitation of σ phase. The R and γ′ phases will also be inhibited when the cooling rate goes up further and only FCC and R phases can be found when the cooling rate reaches up around 80 °C/s. Consequently, the σ phase in Q1 HEA is restrained because of the high cooling rate when quenched in water. The experiments shows that the γ′ phase did not occur in Q1 and AC HEAs (red circles in Fig. 5(c)), illustrating that the accuracy of the CALPHAD method need further improving. As for FC2 and Q2 HEAs, all the phases occurred in the course of heat preservation, thus the cooling rate only affected the volume fraction of phases.

Our results indicated that kinetics of phase transformation plays a crucial role in phase selection of the CoCrFeNiTi_0.4_ HEA. The phases can be controlled using kinetic ways according to the CCT curve. It seems that the sluggish diffusion in HEAs did not affect the kinetic behavior in this alloy system especially in the intermediate temperature. In fact, kinetic effect not only works for the special CoCrFeNiTi_0.4_ HEA but affects the phase selection of all HEAs. Li *et al.*^40^ reported that BCC phase can be obtained by supercooling method in the well-known FCC single phase CoCrFeNi HEA and the mechanical properties of the HEA was enhanced by three times. The rapid solidification behavior of CoCrCuNiFe_x_ HEA was also studied and useful nano-phases were found in the matrix^41^, which would unquestionably strengthen the HEA dramatically. Ravi *et al.*^42^ reported that synthesis route can have a significant influence on the process of phase-choice and -evolution in different HEAs and this is actually a result of kinetic effect. Therefore, kinetic effect is universal in HEAs and controlling phase selection using kinetic ways can be applied to all HEAs. Besides, the very recent excellent publications^30^,^31^ indicated that the multi-phase HEAs have better mechanical performance. Therefore, because the phase compositions can be adjusted using a kinetic way and the better performance of multi-phase HEAs, the high-entropy alloy-design strategy can be applied to a wide range of complex materials, and should not be limited to the goal of creating single-phase solid solutions.

## Conclusions

In summary, the kinetic effect of phase transformation and selection in HEAs are investigated by taking the CoCrFeNiTi_0.4_ HEA as example. The phase selection of CoCrFeNiTi_0.4_ HEA under different process routines was determined. Extremely rapid solidification process inhibited the formation of σ, γ′ and R phases in the CoCrFeNiTi_0.4_ HEA. With the decrease of cooling rate, Laves, R and FCC phases occurred in the sample annealed at 1080 °C and quenched in water. When the cooling rate is slow enough, such as furnace cooling, all the phases showed up in the CoCrFeNiTi_0.4_ HEA. The CALPHAD method was used to calculate the phase compositions and phase transformation kinetics and the calculated results are used to guide the experiment work. The significant influence of the kinetic effect on the phase selection of CoCrFeNiTi_0.4_ HEA indicates that it is possible and important to take the solidification and solid-state phase transformation kinetics into consideration in controlling the phase selection of HEAs for a better performance. Furthermore, with this kinetic methods of tailoring phase compositions proposed here, it is not necessary to limit HEAs to single solid solutions anymore and multi-phase HEAs deserve more attention.

## Additional Information

**How to cite this article**: He, F. *et al.* Kinetic ways of tailoring phases in high entropy alloys. *Sci. Rep.* **6**, 34628; doi: 10.1038/srep34628 (2016).

## Figures and Tables

**Table 1:** Chemical compositions of the CoCrFeNiTi_0.4_ HEA at different states (at. %), as-cast (AC), heat affected zone (HAZ), laser remelted zone (LR), furnace cooling after annealed at 900 °C (FC2).

States	Phases	Co	Cr	Fe	Ni	Ti
AC	FCC	22.52	26.08	26.08	20.72	4.61
	Laves	21.99	22.29	22.19	22.77	10.76
	R	20.17	13.28	14.29	35.87	16.40
HAZ	FCC	20.51	26.21	25.93	22.62	4.73
	Laves	21.99	23.29	22.49	22.52	9.71
LR	FCC	22.48	23.69	23.79	22.13	7.92
	Laves	22.43	24.47	23.67	21.23	8.19
FC2	FCC	25.10	22.70	26.27	19.02	6.91
	Laves	20.00	42.15	25.23	7.65	4.97
	R	22.54	5.29	8.11	43.79	20.27
	σ	24.00	22.16	21.31	22.26	10.28
