# Corrigendum: Kinetic ways of tailoring phases in high entropy alloys

**DOI:** 10.1038/srep46914

**Published:** 2017-12-22

**Authors:** Feng He, Zhijun Wang, Yiyan Li, Qingfeng Wu, Junjie Li, Jincheng Wang, C. T. Liu

Scientific Reports 6: Article number: 34628; 10.1038/srep34628 published online: 09 30 2016; updated: 12 22 2017.

This Article contains an error in the Experimental section.

“Elements of Co, Cr, Fe, Ni and Nb with purity of 99.95% were used as raw materials”.

should read:

“Elements of Co, Cr, Fe, Ni and Ti with purity of 99.95% were used as raw materials”.

